# Transcription of *cis* Antisense Small RNA MtlS in *Vibrio cholerae* Is Regulated by Transcription of Its Target Gene, *mtlA*

**DOI:** 10.1128/JB.00178-19

**Published:** 2019-06-21

**Authors:** Mark G. Zhang, Jane M. Liu

**Affiliations:** a Department of Biology and Biological Engineering, California Institute of Technology, Pasadena, California, USA; b Department of Chemistry, Pomona College, Claremont, California, USA; Princeton University

**Keywords:** *Vibrio cholerae*, antisense, mannitol, small regulatory RNAs, transcriptional interference

## Abstract

Vibrio cholerae, the facultative pathogen responsible for cholera disease, continues to pose a global health burden. Its persistence can be attributed to a flexible genetic tool kit that allows for adaptation to different environments with distinct carbon sources, including the six-carbon sugar alcohol mannitol. V. cholerae takes up mannitol through the transporter protein MtlA, whose production is downregulated at the posttranscriptional level by MtlS, a *cis* antisense small RNA (sRNA) whose promoter lies within the *mtlA* open reading frame. Though it is known that *mtlS* expression is robust under growth conditions lacking mannitol, it has remained elusive as to what factors govern the steady-state levels of MtlS. Here, we show that manipulating *mtlA* transcription is sufficient to drive inverse changes in MtlS levels, likely through transcriptional interference. This work has uncovered a *cis*-acting sRNA whose expression pattern is predominantly controlled by transcription of the sRNA’s target gene.

**IMPORTANCE** Vibrio cholerae is a bacterial pathogen that relies on genetic tools, such as regulatory RNAs, to adapt to changing extracellular conditions. While many studies have focused on how these regulatory RNAs function, fewer have focused on how they are themselves modulated. V. cholerae expresses the noncoding RNA MtlS, which can regulate mannitol transport and use, and here we demonstrate that MtlS levels are controlled by the level of transcription occurring in the antisense direction. Our findings provide a model of regulation describing how bacteria like V. cholerae can modulate the levels of an important regulatory RNA. Our work contributes to knowledge of how bacteria deploy regulatory RNAs as an adaptive mechanism to buffer against environmental flux.

## Abstract (precis)

Vibrio cholerae is a bacterial pathogen that relies on genetic tools, such as regulatory RNAs, to adapt to changing extracellular conditions. While many studies have focused on how these regulatory RNAs function, fewer have focused on how they are themselves modulated. V. cholerae expresses the noncoding RNA MtlS, which can regulate mannitol transport and use, and here we demonstrate that MtlS levels are controlled by the level of transcription occurring in the antisense direction. Our findings provide a model of regulation describing how bacteria like V. cholerae can modulate the levels of an important regulatory RNA. Our work contributes to knowledge of how bacteria deploy regulatory RNAs as an adaptive mechanism to buffer against environmental flux.

## INTRODUCTION

*Vibrio cholerae* is the Gram-negative bacterium responsible for the gastrointestinal ailment cholera, a continuing global health concern that afflicts an estimated 1 million to 4 million people worldwide (1, 2). A facultative pathogen, V. cholerae must adapt to environmental fluctuations both within and between its two primary habitats: the aquatic environment and the human small intestine (3). To buffer against such variation, which can include changes in nutrient availability, salinity, temperature, and acidity, V. cholerae exercises diverse regulatory mechanisms to accordingly alter its gene expression profile (4–8). One such method of genetic regulation entails the production of regulatory small RNAs (sRNAs), short, usually noncoding RNAs that can activate and/or repress the expression of their target genes at the transcriptional and/or posttranscriptional level through an array of distinct mechanisms (9–11). Most often, the sRNAs accomplish this regulation by directly base pairing with their target mRNAs, which can result in translational inhibition, codegradation, or transcript stabilization. In rarer cases, sRNAs can also encode proteins, attenuate transcription, or even directly bind regulatory proteins (11–14). In V. cholerae specifically, sRNAs have been confirmed to play a role in physiological processes, such as virulence, quorum sensing, and biofilm formation (15–18).

sRNAs are typically divided into two categories, *trans* acting or *cis* acting, depending on where the sRNA is transcribed relative to the gene(s) that it regulates (9). *trans*-Acting sRNAs, the more commonly studied of the two types, are transcribed at a genetic locus separate from the gene(s) that they regulate and often function via imperfect base pairing with their target mRNAs. On the other hand, *cis*-acting sRNAs are transcribed from the same genetic locus but in an antisense orientation to the genes that they regulate, resulting in extended regions of perfect complementarity. *cis* antisense RNAs carry the unique advantage of (i) being transcribed proximal to their target, which results in increased effective molarity, and (ii) sharing extended lengths of perfect complementarity to their target, allowing for stronger duplex formation and, thus, tighter regulation (19–21). Although *cis* antisense RNAs have garnered significantly more notice over the past decade, they have received scarce attention compared to their *trans*-acting counterparts (19, 20). At the same time, in one study, 47% of the RNAs transcribed from the V. cholerae genome were antisense transcripts (17). The importance and function of these antisense transcripts, including the *cis*-acting sRNAs, therefore warrant attention.

**FIG 1:**
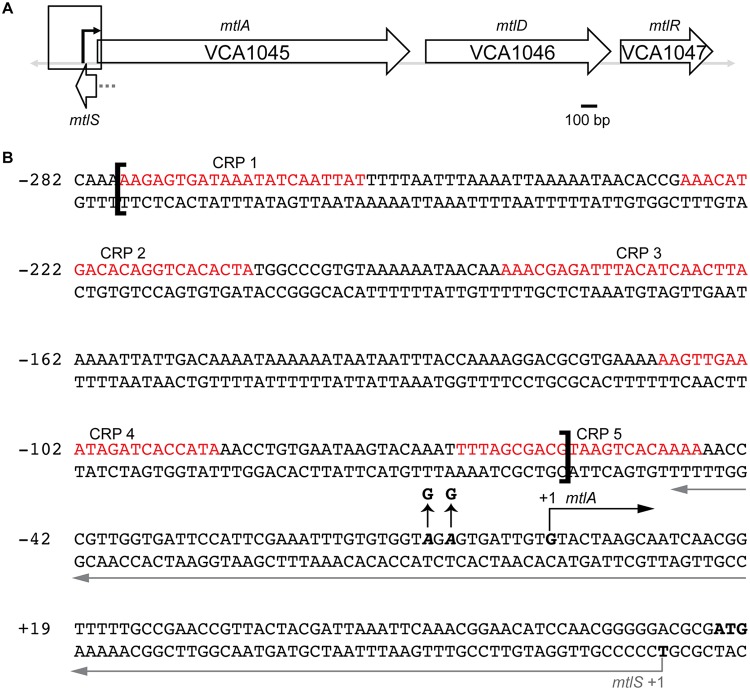
The *mtl* gene locus in V. cholerae. (A) VCA1045, VCA1046, and VCA1047 (*mtlA*, *mtlD*, and *mtlR*, respectively) are three unique genes involved in the transport and/or metabolism of mannitol. MtlS is an antisense sRNA relative to *mtlA* with 71 bp of complementarity to the *mtlA* 5′ UTR. The black arrow marks the +1 site of transcription of *mtlA*. The gray dotted line denotes the putative promoter region of *mtlS*, housed within the *mtlA* coding region. (B) Nucleotide composition of the *mtlA* promoter region and 5′ UTR, as outlined by the box in panel A. The five empirically verified CRP-binding sites are indicated (29). The brackets denote the region excised in the PmtlA_ΔCRPbs strain. The straight black arrows indicate the two A-to-G point mutations in the −10 region of *mtlA* to construct the PmtlA_−10mut strain. The start of transcription of *mtlA* is indicated with a black right-angle arrow. The start of transcription of MtlS is indicated with a gray arrow that continues along the length of MtlS. Numbering is based on the transcription start site of *mtlA* as +1.

MtlS is a 120-nucleotide (nt) *cis* antisense RNA located within the *mtl* locus of V. cholerae which encodes three genes related to the transport and metabolism of mannitol: *mtlA* (encoding the mannitol-specific enzyme IIABC component of the phosphotransferase system [PTS]), *mtlD* (a mannitol-1-phosphate dehydrogenase), and *mtlR* (a transcriptional repressor of *mtlA*) (Fig. 1A) (22–24). Mannitol is one of the most abundant and widely distributed natural sugar alcohols and the primary photosynthetic product of brown algae (25, 26). Genes within the *mtl* locus have been implicated in pathogenically relevant behaviors, including biofilm formation and transitions from the host into the aquatic environment (8, 27, 28), thereby suggesting that mannitol is an important carbon source in the V. cholerae life cycle.

Consistent with the importance of mannitol in the V. cholerae life cycle, at least three regulators collaborate to fine-tune expression of *mtlA*. The global regulator cAMP receptor protein (CRP) is a transcriptional activator of *mtlA* (29). Opposing the activity of CRP, MtlR acts as a transcriptional repressor of *mtlA* (23). Studies concerning the regulation of the *mtl* locus provide a model for maximal *mtlA* transcription that relies on two conditions: high cAMP-CRP activity and low MtlR activity (23, 29). In glucose-containing medium, low cAMP levels preclude *mtlA* from being transcribed. In growth medium excluding mannitol but supplemented with carbon sources, such as mannose, fructose, sucrose, etc., cAMP levels may be sufficiently high, but high MtlR activity prohibits *mtlA* transcription. When mannitol is the sole carbon source, both cAMP-CRP activity is adequately high and MtlR activity is sufficiently low to allow the robust transcription of *mtlA*. However, neither the cAMP-CRP and MtlR interface nor the mechanistic basis behind MtlR repression has been fully defined (23).

The third characterized regulator of *mtlA* is MtlS, which sits in the intergenic region between *mtlA* and VCA1044 (encoding a hypothetical protein), where it shares 71 bp of perfect complementarity with the 5′ untranslated region (UTR) of *mtlA*. As a repressor of *mtlA*, MtlS is expressed abundantly in the absence of mannitol, including during growth in Luria-Bertani (LB) or minimal medium supplemented with a nonmannitol carbon source (30). We recently reported that MtlS represses MltA synthesis at the posttranscriptional level by binding to the 5′ UTR of the *mtlA* mRNA and occluding ribosomal binding (21). However, while the regulatory elements governing *mtlA* expression are relatively well characterized, we have little understanding regarding the factors that control *mtlS* expression.

Several sRNAs have their regulatory basis for expression well characterized. SgrS and OxyS, two of the most comprehensively studied *trans*-acting sRNAs from Escherichia coli, fall under the control of transcriptional regulators SgrR and OxyR, respectively, both of which lie immediately upstream of their cognate sRNAs. These transcriptional regulators respond to the buildup of intermediates related to the physiological stress conditions that the sRNAs help the cell adapt against: SgrR senses the buildup of phosphorylated glycolytic intermediates through an unknown mechanism (31), while OxyR detects oxidative stress through hydrogen peroxide-driven disulfide bond formation that results in structural changes for the protein (32, 33). In V. cholerae, the Qrr sRNAs, which are involved in regulating quorum sensing, are transcribed through the activity of LuxO, a DNA-binding regulator that is activated via phosphorylation when the bacteria are at a low cell density (16). When present, the Qrr sRNAs base pair with the 5′ UTR of *hapR* mRNA, decreasing synthesis of the master transcriptional regulator of quorum sensing. Qrr sRNA levels are also subject to several regulatory feedback loops. In the presence of phosphorylated LuxO, HapR activates transcription of the Qrr sRNAs, presumably minimizing unnecessary synthesis of the master regulator (34). The Qrr sRNAs, furthermore, can also repress the translation of LuxO, ultimately allowing for tight control and fine-tuning of Qrr levels to provide flexible and nuanced regulation of quorum sensing (35).

As for *cis* antisense sRNAs, in Shigella flexneri, RnaG is a 450-nt-long noncoding RNA that negatively affects transcription of *icsA*, encoding a protein required for the invasion of intestine epithelial cells and the intracellular spread of the pathogen (12). RnaG affects *icsA* expression through a combination of transcriptional interference and transcriptional attenuation, and the transcription of RnaG itself is mildly repressed by the nucleoid-associated protein H-NS at low temperatures and the transcriptional regulator VirF at high temperatures (12, 36). As H-NS and VirF also affect *icsA* transcription, the two proteins and RnaG collaborate for the fine-tuned regulation of virulence gene expression by the pathogen. In Salmonella enterica serovar Typhimurium, transcription of the 1.2-kb antisense RNA AmgR is activated by the two-component regulatory system PhoP/PhoQ in response to low Mg^2+^ concentrations (37). Although longer than a typical sRNA, AmgR effectively downregulates the synthesis of MgtB and MgtC, which are involved in Mg^2+^ transport and virulence in mice, respectively. However, it is important to keep in mind that a majority of the regulatory RNAs whose basis for expression is well explored, including SgrS, the Qrr sRNAs, and AmgR, share the feature of having promoters that do not lie in the open reading frame of another gene. A number of *cis* antisense RNAs, including MtlS from V. cholerae, are transcribed from promoters that overlap extensively, if not completely, the coding region of the very genes that they regulate (12, 30, 38–41), which can complicate dissection of their transcriptional regulation. Indeed, most of these sRNAs are particularly poorly understood when it comes to the regulation behind their expression.

MtlS exhibits a carbon source-dependent expression profile that logically aligns with its function as a repressor of mannitol utilization. V. cholerae produces nearly undetectable amounts of MtlS under conditions where mannitol is the sole carbon source but synthesizes robust levels of MtlS under growth conditions without any mannitol present (30). We set out to determine the mechanistic foundation underpinning this pattern. Here, we report that the transcription of MtlS is controlled primarily by the extent of *mtlA* transcription occurring in the antisense direction. Rather than utilizing its own promoter as the basis for sugar-dependent expression, *mtlS* instead predominantly relies on regulatory activity at the *mtlA* promoter. Our analysis points toward transcriptional interference as the likely mechanism of action in the regulation of MtlS levels. Our findings reveal a method of controlling the expression of a *cis* antisense regulatory small RNA, whereby transcription from the opposite antisense gene controls sRNA levels.

## RESULTS

### Transcription of *mtlS* and *mtlA* is inversely coupled.

**FIG 2:**
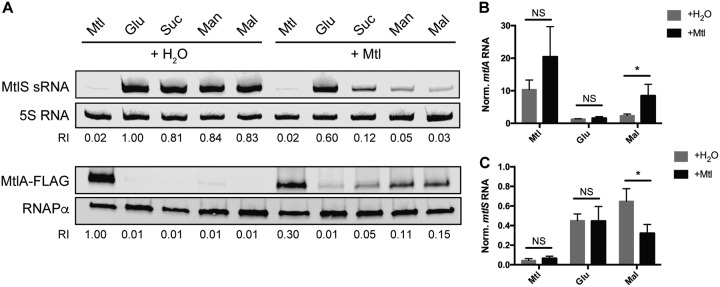
Mannitol addition concurrently increases *mtlA* expression and decreases *mtlS* expression. V. cholerae was grown to mid-log phase in minimal medium with 0.4% (wt/vol) mannitol (Mtl), glucose (Glu), sucrose (Suc), mannose (Man), or maltose (Mal) supplemented with an additional 0.4% mannitol (+Mtl) or an equal volume of water (+H_2_O). (A) Cell lysates were subjected to both Northern blot analysis (for MtlS) and Western blot analysis (for MtlA). The relative intensity (RI) of each sample compared to the intensity of glucose plus H_2_O (for MtlS analysis) or mannitol plus H_2_O (for MtlA analysis) is shown beneath each band. Blots are representative of those from at least two independent experiments. (B, C) Total RNA was used for qRT-PCR analysis with primers specific to *mtlA* (B) or *mtlS* (C). The levels of *mtlA* and MtlS RNA were normalized (Norm.) to those of an endogenous 4.5S RNA control. Reported are the means and standard deviations from three biological replicates. *P* values are based on two-tailed unpaired *t* test. *, *P < *0.05; NS, not significant.

We set out to determine how V. cholerae exerts control over MtlS levels, producing the sRNA only when necessary to repress expression of *mtlA*. Given that MtlS sRNA levels in V. cholerae are high under all tested growth conditions lacking mannitol but barely detectable when cells are grown in minimal medium supplemented with only mannitol, we speculated whether mannitol played a role in repressing MtlS levels. To test this question, we grew V. cholerae in minimal medium supplemented with a carbon source, in addition to either mannitol or water (Fig. 2). We chose to use mannitol, glucose, sucrose, and mannose as representative PTS sugars (sugars whose transport depends entirely on the PTS) (42) and maltose as a representative non-PTS sugar in order to assess whether the observed phenomena were specific to the PTS system. Northern blot analysis for MtlS indicated that the addition of mannitol is sufficient to decrease MtlS sRNA levels (Fig. 2A). Paired with glucose, mannitol led to only a minor decrease in MtlS. However, when paired with a sugar such as mannose or maltose, the addition of mannitol to the growth medium was sufficient to decrease MtlS levels over 90% compared to those for the control, in which only H_2_O was added to the base carbon source.

We then postulated potential conduits through which mannitol could decrease MtlS levels. We turned our attention to *mtlA*, since MtlA protein levels inversely mirror the expression profile of MtlS (i.e., the MtlA protein is most abundant when cells are provided with mannitol as the sole carbon source). Consequently, we questioned whether mannitol could also be increasing MtlA levels, even when another suitable carbon source is present. We took the same cell samples that we grew in preparation for the MtlS Northern blot analysis and simultaneously used them to probe MtlA levels (Fig. 2A). We saw a precise inverse trend compared to what we observed for MtlS. That is, the addition of mannitol upregulated the synthesis of MtlA, and the extent to which it activated *mtlA* was strictly dependent on the accompanying carbon source. As is the case for MtlS, mannitol had almost no effect when it was paired with glucose but upregulated *mtlA* expression when it was present in conjunction with sucrose, mannose, or maltose.

The unique ability of glucose to suppress mannitol’s capacity to affect MtlA levels is likely due to carbon catabolite repression, a phenomenon that describes how a preferable sugar, such as glucose, can repress the transcription of genes related to the transport and metabolism of other, less favorable sugars (43, 44). Glucose inhibits CRP activity by way of downregulating the production of its ligand, cAMP (45, 46). *mtlA* requires CRP for transcription (29), and it is reasonable to speculate that the addition of mannitol to medium already containing glucose is insufficient to stimulate the transcription of *mtlA* since CRP remains inactive.

Our previous investigations into *mtlA* mRNA levels focused on growth in minimal medium supplemented with a single carbon source. Thus, we also evaluated the effect on *mtlA* mRNA upon adding mannitol to growth medium containing another carbon source. We conducted quantitative reverse transcription-PCR (qRT-PCR) with primers specific to *mtlA* using total RNA extracted from V. cholerae grown under the same conditions previously described with maltose as our representative nonmannitol, nonglucose carbon source (Fig. 2B). We observed that cells grown in minimal medium supplemented with both maltose and mannitol had nearly triple the amount of *mtlA* mRNA as cells grown in medium containing maltose only (Fig. 2B; compare the gray and black bars for maltose [Mal]), indicating that mannitol is able to increase *mtlA* mRNA levels in the presence of maltose. We also noted that, in line with the Western blotting data, mannitol addition was insufficient to upregulate *mtlA* RNA levels when paired with glucose (compare the gray and black bars for glucose [Glu] in Fig. 2B and compare Fig. 2A and B). Doubling of the amount of mannitol in the growth medium also did not have a significant impact on *mtlA* mRNA levels (Fig. 2B; compare the gray and black bars for mannitol [Mtl]). Using the same RNA samples, we also performed qRT-PCR using primers specific to *mtlS* (Fig. 2C) in order to evaluate the reproducibility of the trends observed from the MtlS Northern blot analysis. We saw that the addition of mannitol significantly decreased MtlS levels under maltose growth conditions but had no significant effect under glucose or mannitol base conditions (Fig. 2C; compare the gray and black bars), both of which largely align with the conclusions drawn from Northern blot analysis (compare Fig. 2A and C). These data collectively demonstrate two things: (i) the addition of mannitol can simultaneously increase *mtlA* mRNA levels and decrease MtlS levels, depending on the accompanying carbon source, and (ii) *mtlA* and MtlS RNA levels are precisely coupled: the amount by which *mtlA* mRNA levels increase as a result of mannitol addition accurately informs the extent to which MtlS levels decrease.

### Mannitol can activate the *mtlA* promoter but does not affect activity at the *mtlS* promoter.

To dissect the mechanistic basis behind the above-described observations, we sought to determine whether the *mtlA* promoter or the *mtlS* promoter (or both) was sensitive to growth conditions in which mannitol is present. Specifically, we evaluated the validity of three scenarios when mannitol is added to the growth medium: (i) mannitol activates transcription from the *mtlA* promoter while also repressing transcription from the *mtlS* promoter; (ii) mannitol activates transcription only from the *mtlA* promoter, which subsequently and indirectly results in lowered MtlS levels; and (iii) mannitol represses transcription only from the *mtlS* promoter, which indirectly results in increased *mtlA* mRNA levels. We reasoned that in the last two scenarios, such sequential regulation might arise due to factors such as transcriptional interference and codegradation, both of which have been associated with several *cis* antisense RNAs and their targets (19). Transcriptional interference postulates that when two convergent promoters are spaced sufficiently close together, such as in the case of *mtlA* and *mtlS* (Fig. 1), the expression of one gene can interfere with transcriptional read-through from the opposite promoter (47–49). Codegradation can occur when two RNAs form a duplex that results in the rapid, RNase-mediated degradation of both transcripts (50, 51).

To distinguish among the three possibilities, we pursued a LacZ reporter-based approach to uncouple transcription between the *mtlA* promoter and the *mtlS* promoter. We fused the region directly upstream of the transcription start site (+1) for either *mtlA* or *mtlS* with the E. coli *lacZ* gene and inserted the construct in a neutral locus within the V. cholerae genome. We previously mapped the transcription start sites of both *mtlA* and *mtlS* (22, 30). Using the PromoterHunter tool, we identified putative −10 and −35 elements that precede the +1 site of *mtlS* (52); the presence of additional regulatory sequences, however, has not been investigated. Therefore, to ensure that we captured all essential promoter elements, we used the 500 bp upstream of the *mtlS* transcription start site to construct the *mtlS-lacZ* fusion. For consistency, we also used the 500 bp upstream of our *mtlA* reporter, knowing that this fragment would include all empirically verified regulatory regions, such as the five essential activating CRP-binding sites (Fig. 1B) (29).

**FIG 3:**
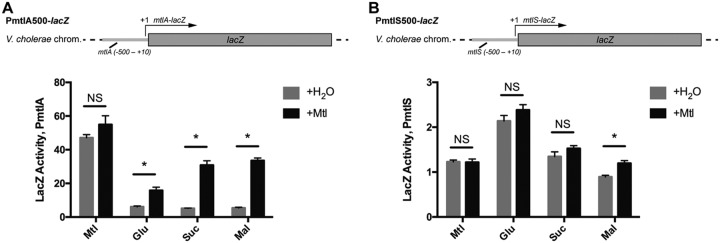
LacZ reporter constructs uncouple transcription between *mtlA* and *mtlS*. V. cholerae strains harboring *lacZ* transcriptional fusions to the 500 bp upstream of the +1 site of *mtlA* (A) or *mtlS* (B) were grown to late log phase in minimal medium supplemented with 0.4% the indicated sugar along with an additional 0.4% (wt/vol) mannitol (+Mtl) or an equal volume of H_2_O. LacZ activity is reported as the average increase in the OD_420_ over the course of the assay normalized to the OD_600_ (mean OD_420_ per minute per OD_600_). Reported are the means and standard deviation from 4 biological replicates. *, statistical analysis indicates that the results of supplementation with H_2_O versus mannitol are true discoveries (the false-discovery rate *q* value was set to 1%); NS, not significant. All results shown are representative of those from at least two independent experiments.

We grew the *mtlA* and *mtlS* reporter strains (PmtlA500-*lacZ* and PmtlS500-*lacZ*, respectively) in minimal medium supplemented with a single carbon source, in addition to either water or mannitol, again choosing several PTS sugars (mannitol, glucose, and sucrose) and one representative non-PTS sugar, maltose. We then performed LacZ assays in order to determine how transcription from each of the promoters behaved independently of a proximally located antisense promoter (Fig. 3). The PmtlA500-*lacZ* strain displayed a pattern of *lacZ* expression in a manner nearly identical to that observed for endogenous *mtlA* through Western blot and qRT-PCR analyses (compare Fig. 2A and B and Fig. 3A). For growth conditions supplemented with a sole carbon source (Fig. 3A, gray bars), LacZ activity was the highest in medium containing strictly mannitol. Moreover, addition of mannitol to the growth medium significantly increased reporter activity in a sugar-dependent manner, with the increase being most pronounced under maltose-containing growth conditions (compare the differences between the gray and black bars in Fig. 3A). However, the PmtlS500-*lacZ* strain demonstrated an activity profile that deviated from what was observed for MtlS through Northern blot and qRT-PCR analyses (compare Fig. 2A and C and Fig. 3B). Reporter activity reflecting MtlS transcription was not consistently high during growth in medium supplemented with nonmannitol sugars, nor was it particularly low in medium supplemented strictly with mannitol (compare the gray bars in Fig. 3B). The reporter activity from the PmtlS500-*lacZ* strain was elevated when cells were grown in medium supplemented with glucose, indicating that the sugar may be able to modestly effect the direct upregulation at the *mtlS* promoter. Importantly, the addition of mannitol to the growth medium had no significant effect on reporter activity in medium supplemented with mannitol, glucose, or sucrose (compare the differences between the gray and black bars in Fig. 3B). In medium supplemented with maltose, the addition of mannitol actually led to a small but significant increase in reporter activity. These results demonstrate that the addition of mannitol to the growth medium does not affect transcriptional activity from the *mtlS* promoter in a manner consistent with the observed MtlS levels. Considering, too, that our PmtlA500-*lacZ* reporter behaves most consistently with what we observed with endogenous *mtlA* expression, our LacZ reporter assay data point toward *mtlA* as the pivotal center of regulation at the *mtlA-mtlS* locus (the second scenario described above): the addition of mannitol is able to activate transcription from the *mtlA* promoter. However, it remains to be demonstrated whether activation of *mtlA* was sufficient to repress MtlS levels.

### Manipulating *mtlA* transcription results in inverse changes in MtlS levels.

**FIG 4:**
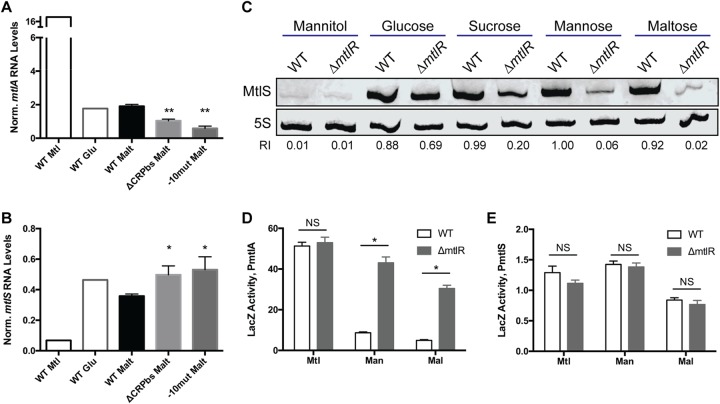
Manipulating *mtlA* expression results in corresponding inverse changes in MtlS levels. V. cholerae strains were grown to mid-log (A, B, C) or late log (D, E) phase in minimal medium supplemented with the indicated carbon source. (A, B) The V. cholerae *mtlA* promoter region was ablated either by deleting the five CRP-binding sites within the promoter (ΔCRPbs) or by creating two point mutations in the −10 promoter region (−10mut). Total RNAs from these strains were used for qRT-PCR analysis with primers specific to *mtlA* (A) or *mtlS* (B). The levels of *mtlA* and MtlS RNA were normalized to those of an endogenous 4.5S RNA control. Reported are the means and standard deviations from three biological replicates (except for mannitol and glucose, where *n* = 1). *P* values are based on a two-tailed unpaired *t* test comparing the mutant to the wild type (WT). *, *P < *0.05; **, *P < *0.01. (C) Total RNAs from the V. cholerae wild-type or Δ*mtlR* strain were used for Northern blot analysis. The relative intensity (RI) of each sample compared to the intensity of the mannose wild type is shown underneath each band. (D, E) Cell lysates from the wild type and the Δ*mtlR* mutant of V. cholerae strains harboring *lacZ* transcriptional fusions to the 500 bp upstream of the +1 site of *mtlA* (D) or *mtlS* (E) were used for LacZ assays, as described in the legend to Fig. 3. Reported are the means and standard deviation from 4 biological replicates. *, statistical analysis indicates that wild type versus Δ*mtlR* strain are true discoveries (the false-discovery rate *q* value was set to 1%); NS, not significant. All results shown are representative of those from at least two independent experiments.

We assessed the validity of a regulatory model centered on *mtlA* by directly manipulating expression at the *mtlA* promoter to see if we could drive the corresponding inverse changes in *mtlS* expression. We first constructed two strains harboring mutations in the *mtlA* promoter region (Fig. 1B). The first strain lacked the region that contains the five CRP-binding sites. These five binding sites were previously shown to be essential for activation of the *mtlA* promoter (29). As the fifth CRP-binding site overlaps the 3′ end of *mtlS*, we included half of this CRP-binding site to preserve the integrity of *mtlS*. The second strain that we constructed contained two point mutations in the expected −10 promoter region of *mtlA*. Confirming the abrogation of *mtlA* expression in these mutants, neither of the two strains could grow in medium in which mannitol was the only carbon source (data not shown). We grew these promoter mutants in medium supplemented with maltose and conducted qRT-PCR with primers specific to *mtlA* and *mtlS* (Fig. 4A and B). We observed similar results in both strains: *mtlA* mRNA levels decreased significantly compared to wild-type levels, while MtlS levels were upregulated relative to those in the wild type. These results further confirm that our mutations successfully obstructed transcription from the *mtlA* promoter and imply that such obstruction was sufficient to increase MtlS levels. It is important to note that we performed these experiments in maltose-containing medium, a representative growth condition associated with the nearly absent production of MtlA and the abundant production of MtlS in wild-type V. cholerae (Fig. 2A). Thus, even under an *mtlA*-repressive condition, *mtlA* is not fully off, nor is *mtlS* fully on, since manipulations could still be made to further decrease or increase RNA levels, respectively.

While this promoter-ablation approach demonstrated that decreasing *mtlA* expression could increase *mtlS* expression, we also sought the opposite approach and determined whether increasing *mtlA* mRNA levels could lower MtlS levels. To accomplish this, we used a strain with an in-frame deletion of *mtlR*, which encodes a transcriptional repressor of *mtlA*; compared to the wild type, strains lacking MtlR have higher levels of *mtlA* mRNA and MtlA protein when grown in minimal medium with glucose, maltose, or mannose as the sole carbon source (23). We previously reported that MtlR repression of *mtlA* depends on the supplemented carbon source. Medium containing only mannitol results in no observable repression by MtlR, and medium containing only glucose results in low levels of repression, while medium supplemented with only mannose or maltose results in the highest levels of repression (23). Consistent with these previous observations, Northern blot analysis indicated that deletion of *mtlR* lowers MtlS levels in a sugar-dependent manner (Fig. 4C). Deletion of *mtlR* had a minor effect on MtlS levels when cells were grown in minimal medium supplemented with glucose but resulted in pronounced downregulation when cells were grown with sugars such as mannose and maltose. These results support a model in which activation of *mtlA* transcription can result in decreased MtlS levels.

We did, however, question whether MtlR might affect MtlS levels directly by acting on the *mtlS* promoter. To address this, we created an in-frame deletion of *mtlR* in both our PmtlA500-*lacZ* and PmtlS500-*lacZ* reporter strains and grew the cells in minimal medium supplemented with various carbon sources (Fig. 4D and E). We observed that deleting *mtlR* did, as predicted, increase LacZ activity from the PmtlA500-*lacZ* strain under growth conditions supplemented with a nonmannitol, nonglucose sugar. However, the lack of MtlR had no effect on LacZ activity in the PmtlS500-*lacZ* strain, regardless of the growth medium. These data establish MtlR as an indirect activator of MtlS transcription by virtue of being a transcriptional repressor of *mtlA*. The extent to which MtlR is a repressor of *mtlA* transcription reflects the extent to which MtlR is an indirect activator of *mtlS*. Overall, these observations point toward a regulatory model whereby expression of *mtlS* is dictated by transcriptional activity from the *mtlA* locus. Moreover, the LacZ activity from the PmtlS500-*lacZ* strain, under all conditions tested, was quite low (compare Fig. 3A and B and Fig. 4D and E); the *mtlS* promoter may be fairly weak, particularly in comparison to the *mtlA* promoter. These observations point toward transcriptional interference as a likely mechanism by which MtlS levels are regulated: transcription from the strong *mtlA* promoter inhibits transcription from the weaker *mtlS* promoter.

### *mtlA*-mediated regulation of *mtlS* does not depend on codegradation.

**FIG 5:**
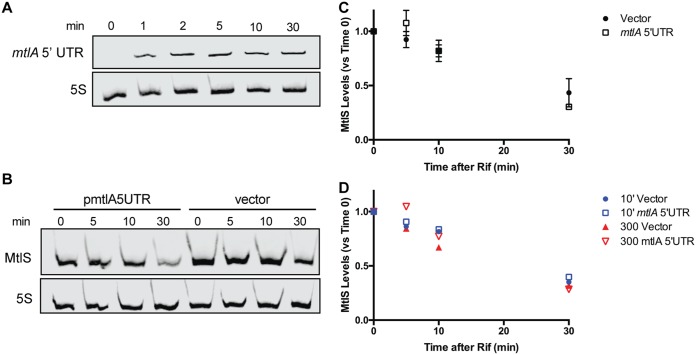
Ectopic expression of the 5′ UTR of *mtlA* does not affect the stability of MtlS. (A) V. cholerae harboring pmtlA5UTR was grown in minimal medium supplemented with 0.4% (wt/vol) maltose to mid-log phase, whereupon an aliquot was taken (0 min). The remaining cells were induced with 0.02% arabinose and aliquots were taken at the indicated times. (B) V. cholerae harboring pmtlA5UTR or a vector control were grown as described in the legend to panel A and induced with 0.02% arabinose. After 2 min, the cells were treated with 200 μg/ml rifampin (Rif) and aliquots were taken at the indicated times. Total RNA was used for all Northern blots, and 5S RNA was used as a loading control. (C) Quantification analysis of the Northern blot from panel B and two additional independent experiments. MtlS signals were normalized to the 5S RNA loading control and are reported as a percentage of the value at time zero for each respective strain. Shown are the mean and standard deviation for each time point. (D) Quantification analysis of Northern blots carried out as described in the legend to panel B but with either 300-μg/ml rifampin treatment or a 10-min induction with arabinose prior to treatment with rifampin.

Although the data presented above support a model in which the transcription of *mtlA* represses MtlS levels via transcriptional interference, we also considered codegradation to be a possible mechanism responsible for the *mtlA*-mediated regulation of *mtlS*. That is, we speculated that some of the *mtlA* mRNA transcribed under mannitol-inducing conditions could be sacrificed to pair with and direct the degradation of MtlS sRNAs, resulting in the lowered levels of MtlS observed in the presence of mannitol. To test this model, we used a V. cholerae strain harboring a plasmid that expresses the 5′ UTR of *mtlA* from an arabinose-inducible plasmid (pmtlA5UTR). This strain was grown in minimal medium supplemented with maltose, conditions in which MtlS levels are high and *mtlA* transcription is low. The addition of arabinose (0.02%) to the growth medium resulted in high levels of the *mtlA* 5′ UTR transcript within the first 2 min of induction (Fig. 5A).

We then determined the half-life for MtlS with or without the presence of the ectopically expressed *mtlA* 5′ UTR transcript. V. cholerae was grown in maltose medium to mid-exponential phase, at which point arabinose was added to induce expression of the *mtlA* 5′ UTR transcript. After 2 min of induction, the transcriptional inhibitor rifampin was added. MtlS levels, normalized to the level of the 5S loading control, were assessed by Northern blot analysis before and after the addition of rifampin. At each of the analyzed time points, the levels of MtlS remaining compared to the levels at time zero were similar in both the control and the strain ectopically expressing the *mtlA* 5′ UTR (Fig. 5B and C). These results indicate that the addition of the *mtlA* 5′ UTR transcript does not negatively impact the stability of MtlS. Neither increasing the amount of rifampin used (300 μg/ml versus 200 μg/ml) nor increasing the time between induction of *mtlA* 5′ UTR transcription and addition of rifampin (10 min versus 2 min) affected the results: MtlS levels decreased similarly over the experimental time frame in all cases (Fig. 5D). These data led us to conclude that the repressive effects of *mtlA* transcription on MtlS levels are not due to the codegradation of the two transcripts. At the same time, we consistently noted that the strain harboring pmtlA5UTR had lower levels of MtlS than the vector control, even after only a brief induction with arabinose (compare the first and fifth lanes in Fig. 5B). We speculate that the induced ectopic expression of the *mtlA* 5′ UTR from a multicopy plasmid may have decreased transcription from the weak, endogenous *mtlS* promoter. Alternatively, the high levels of the *mtlA* 5′ UTR may cause transcriptional attenuation of the sRNA.

## DISCUSSION

The current paradigm in the sRNA field reflects a tendency for sRNAs to have their regulatory functions comprehensively defined but their molecular basis for expression underexplored (11, 53). While it is clear that sRNAs play an integral regulatory role by helping bacteria respond to changes in environmental conditions, precisely how sRNAs are transcribed in response to said changes remains substantially less clear. Thus, further studies aimed at dissecting the pathways that govern sRNA levels will be pivotal toward expanding our knowledge of the functional landscape of sRNA-mediated regulation.

In this study, we provide evidence for a regulatory model detailing the expression pattern of MtlS, a *cis* antisense RNA from V. cholerae whose function as a repressor of *mtlA* has been well defined but whose origin of regulation has yet to be dissected. Here, we report that *mtlS* expression is modulated by the level of transcription occurring from the antisense gene *mtlA*. This paradigm has MtlS not expressed in response to an environmental stimulus; instead, MtlS levels are adjusted based on the amount of *mtlA* being transcribed. In the canonical model of sRNA-mediated gene expression, an environmental stimulus (e.g., temperature, oxidative stress, toxic by-product buildup) signals modulations in sRNA levels that result in the regulation of downstream genetic targets. According to this model, the sRNA acts as an intermediary messenger that relays environmental cues into appropriate changes in gene expression. However, our findings demonstrate that MtlS does not appropriately fit into this mold since the regulation of MtlS levels largely occurs downstream of initial changes in target gene expression. We propose an alternative model that better accounts for MtlS as a secondary regulator. In this model, an environmental cue results in the regulation of a target gene independently of the associated sRNA. Since expression of the sRNA gene is intrinsically linked to that of the target gene, sRNA levels subsequently change. This sRNA can then go on to affect the expression of further downstream targets, which can include the very target gene that the sRNA initially responded to. In the case of MtlS, the availability of mannitol alters the transcription of the target gene *mtlA*, which then affects the levels of MtlS, offering further nuanced regulation of *mtlA* and potentially other targets as well (J. M. Liu, unpublished data).

Like MtlS, transcription of the *cis*-acting RnaG is negatively affected by the transcription of its antisense target, *icsA* (12). The RnaG promoter flanks the start codon of *icsA*, with the −35 hexamer being positioned within the coding sequence of *icsA*. The resulting RnaG transcript is complementary to the first 120 nt of the *icsA* mRNA. In this arrangement, transcription from either promoter (each of which is regulated by known transcription factors) results in inhibition of transcription from the other through a transcriptional interference mechanism. In the case of RnaG and *icsA*, however, it is the sRNA which possesses the strong dominant promoter that dramatically inhibits transcription from the *icsA* promoter (12, 36). In contrast to MtlS and RnaG, the levels of the *cis* antisense RNA SymR remain constant, even when its target, *symE* mRNA, increases in concentration in response to DNA damage (38). Thus, it is evident that not all *cis* antisense RNAs are regulated alike. What all these examples do have in common, however, is that in each case the target of the sRNA is under multimodal regulation involving multiple proteins, in addition to the associated *cis*-acting RNA, allowing for the fine-tuned and tight regulation of gene expression.

While we were unable to ascertain the precise mechanisms by which *mtlA* downregulates MtlS levels, our data and recent literature would suggest that transcriptional interference is the likely candidate in this *cis* antisense system (47). Transcriptional interference has been postulated to manifest in three various forms, promoter occlusion, collision, or sitting duck (47, 54), depending on factors such as the spacing and relative strength of the two promoters. Our LacZ reporter assays suggest that the *mtlS* promoter could be up to 40 times weaker, depending on the growth conditions (Fig. 3). Such asymmetry in promoter strengths could result in promoter occlusion, a phenomenon that relies heavily on an RNA polymerase (RNAP) born from an aggressive promoter passing over a sensitive promoter and inhibiting access. However, since the *mtlA* and *mtlS* promoters are spaced closely together at <100 nt apart, the *mtlA*-MtlS system may instead be subject to sitting duck interference, which describes a collision event whereby an elongating polymerase removes, via collision, an opposing polymerase bound in an open complex (54). At the same time, expression of the *mtlA* 5′ UTR in *trans* was able to reduce MtlS levels without affecting the stability of the sRNA (Fig. 5). We therefore cannot rule out transcription attenuation as a model by which *mtlA* regulates MtlS, particularly when *mtlA* 5′ UTR levels are very high; future efforts will focus on teasing apart the contributions of transcriptional interference and attenuation on MtlS levels. Also, while MtlS levels appear to be mostly governed by the transcription of *mtlA*, there is evidence that additional factors may affect *mtlS*. Although the overall levels of LacZ resulting from the PmtlS500-*lacZ* construct were quite low, LacZ activity was consistently higher in glucose medium and generally lower in maltose medium. Thus, it remains to be seen whether environmental stimuli further contribute to the nuanced control of the levels of the sRNA.

The *mtlA*-MtlS system offers a unique regulatory advantage to an organism that requires tight control over the transport and metabolism of mannitol. In its natural aquatic environments, V. cholerae likely comes across distinct compositions of carbon sources, where mannitol concentrations can range up to 700 μM (3, 55). Our data reveal that MtlS constitutes part of a molecular tool kit that helps V. cholerae respond to these distinct environments and make the appropriate genetic decision regarding the expression of mannitol-related genes. We note that the addition of mannitol to the growth medium stimulated *mtlA* expression to various degrees depending on the accompanying carbon source, with stimulation being nearly undetectable in the case of glucose (Fig. 2). Thus, a high mannitol concentration is insufficient for V. cholerae to activate expression of the *mtl* genes. Rather, mannitol needs to be a preferred carbon source in the context of other accompanying carbon sources.

We purport that MtlS serves as a stringent brake that limits expression of *mtlA*, reserving full expression only for conditions in which mannitol utilization is metabolically favored. In a given environment, mannitol must be preferred for V. cholerae to not only stimulate expression from the *mtlA* promoter but also produce enough *mtlA* mRNA to downregulate MtlS levels through transcriptional interference or attenuation. MtlS thus raises the threshold for what qualifies as a sufficiently mannitol-rich environment for V. cholerae to devote energy toward the costly expression of *mtlA*. Although E. coli possess a *bona fide mtl* operon, it lacks a detectable antisense RNA equivalent to that of V. cholerae (22), implying that V. cholerae has evolved the MtlS sRNA through its own evolutionary lineage to better adapt to changes in extracellular mannitol. Our observations are consistent with a scenario where V. cholerae evolved MtlS through mutations in the *mtlA* coding region that both preserved *mtlA* functionality and produced a viable promoter, in addition to a viable terminator region, within the antisense strand (56). Through this process, V. cholerae would have gained access to a repressive *cis* antisense sRNA while avoiding the need for a separate set of regulatory mechanics to govern MtlS levels, since *mtlS* regulation would be inherently coupled to that of its target antisense gene. While details remain to be fleshed out, our studies support this model for the regulation of the MtlS *cis* antisense RNA, and we are eager to discover whether more *cis* antisense RNAs fit a similar mold.

## MATERIALS AND METHODS

### Bacterial strains, plasmids, and culture conditions.

All plasmids and strains used in this study can be found in Table 1. All primers used in this study can be found in Table 2. The wild-type V. cholerae strain used in this study, from which all subsequent strains were constructed, was the O1 biovar El Tor N16961 Δ*tcpA* strain. This strain was used for safety purposes and is highly attenuated for virulence (57), but it still exhibits phenotypes identical to those of the original wild-type strain N16961 with respect to *mtlS* and *mtlA* expression.

**TABLE 1:** Strains and plasmids used in this study

Strain or plasmid	Description or genotype^*a*^	Reference or source
Strains		
V. cholerae		
JL2	N16961 Δ*tcpA mtlA*-FLAG Sm^r^	Laboratory strain
JL55	N16961 Δ*tcpA mtlA*-FLAG Δ*mtlR* Sm^r^	23
JL142	N16961 Δ*tcpA mtlA*-FLAG/pJML01 Sm^r^ Ap^r^	30
JL463	N16961 Δ*tcpA mtlA*-FLAG PmtlA_ΔCRPbs Sm^r^	This study
JL467	N16961 Δ*tcpA mtlA*-FLAG PmtlA_−10mut Sm^r^	This study
JL494	N16961 Δ*tcpA mtlA*-FLAG ΔVC2338 (−235) Sm^r^	This study
JL495	N16961 Δ*tcpA mtlA*-FLAG ΔVC2338 (−235) PmtlA500-*lacZ*(*Ec*) Sm^r^	This study
JL499	N16961 Δ*tcpA mtlA*-FLAG ΔVC2338 (−235) PmtlS500-*lacZ*(*Ec*); Sm^r^	This study
JL546	N16961 Δ*tcpA mtlA*-FLAG/pmtlA5UTR Sm^r^ Ap^r^	This study
E. coli		
DH5α	F^−^ Δ(*lacZYA-argF*)*U169 recA1 endA1 hsdR17 supE44 thi-1 gyrA96 relA1*	Laboratory strain
DH5αλpir	F^−^ Δ(*lacZYA-argF*)*U169 recA1 endA1 hsdR17 supE44 thi-1 gyrA96 relA1* λ::*pir*	Laboratory strain
SM10λpir	*thi recA thr leu tonA lacY supE* RP4-2-Tc::Mu λ::*pir*	Laboratory strain
TOP10	F^–^ *mcr*A Δ(*mrr-hsdRMS-mcrBC*) ϕ80*lacZ*ΔM15 Δ*lacX74 recA1 araD139* Δ(*ara leu*)*7697 galU galK rpsL endA1 nupG* Sm^r^	Invitrogen
Plasmids		
pCVD442	*oriR6K mobRP4 sacB* Ap^r^	58
pJML01	pBAD24 derivative with +1 start site of transcription after NheI site; Ap^r^	22
pmtlA5UTR	pBAD24 derivative that expresses the entire 5′ UTR of *mtlA*; Ap^r^	This study
pJL1	pCVD442 derivative with 2.2-kb HpaI-digested VC2338 (V. cholerae *lacZ*) cloned into SmaI site of pCVD442; Ap^r^	59
pJL1::*lacZ*(*Ec*)	pJL1 derivative with RBS and coding region of E. coli *lacZ* inserted into the VC2338 fragment of pJL1 in an antisense orientation; Ap^r^	This study

a Sm^r^, streptomycin resistance; Ap^r^, ampicillin resistance.

**TABLE 2:** Primers and probes used in this study

Purpose and primer or probe^*a*^	Sequence (5′→3′)^*b*^
Northern blotting	
IR800-5S	IRD800-CTG TTT CGT TTC ACT TCT GAG TTC GGG ATG GAA
T7 mtlSfor	GGA TCC TAA TAC GAC TCA CTA TAG GGA AAA ACC CGT TGG TGA TTC CAT TCG
T7 mtlSrev	TCC CCC GTT GGA TGT TCC G
T7 mtlA5UTRfor	GGA TCC TAA TAC GAC TCA CTA TAG GGT CCC CCG TTG GAT GTT CCG
T7 mtlA5UTRrev	AAA AAC CCG TTG GTG ATT CCA TTC G
qRT-PCR	
*mtlS*-FW	TCC CCC GTT GGA TGT TCC G
*mtlS*-RV	CCG TTG GTG ATT CCA TTC G
*mtlA*-FW	GGT TAT GCC GAA TAT TGG CGC
*mtlA*-RV	ATA GGC CCA ACC AAA GAG GC
4.5S-FW	CTG GTC CTC CCG CAA CAC
4.5S-RV	GAG ACC CCA GCC ACA TC
Cloning of V. cholerae ΔVC2338 (−235)	
LIU515 (F1)	GCC AAG CTT GCA TGC CGC AAC CGC AGT CAG AAC AC
LIU516 (R1)	CTC TAC GGC GTA CAT TCG GAG TTG TTC TGC GCT TTG AC
LIU517 (F2)	GCA GAA CAA CTC CGA ATG TAC GCC GTA GAG CAA AGG C
LIU518 (R2)	AGT GAA TTC GAG CTC GAC CAT TGC ACC ACA GAT GAA ATG
LIU519 (pCVD_F)	TGT GGT GCA ATG GTC GAG CTC GAA TTC ACT GGC CGT
LIU520 (pCVD_R)	CTG ACT GCG GTT GCG GCA TGC AAG CTT GGC GTA ATC ATG
LIU521 (F0)	CTT GCT CGC TAA CCC AGC G
Cloning of plasmid pJL1::*lacZ*(*Ec*)	
LIU122 (rev vector)	TGT TTC CTG TGT GAA AAA TCA TCA CGC CAT GTA TCA GTG G
LIU123 (fwd vector)	CTG GTG TCA AAA ATA ATA AAA TCC CCG ATT CAT TGC CGA GC
LIU124 (fwd insert)	CAT GGC GTG ATG ATT TTT CAC ACA GGA AAC AGC TAT GAC C
LIU125 (rev insert)	CAA TGA ATC GGG GAT TTT ATT ATT TTT GAC ACC AGA CCA ACT GG
Cloning of V. cholerae PmtlA500-*lacZ*(*Ec*)	
LIU522 (fwd insert)	CAT GGC GTG ATG ATT CAT TTC TTC ATC TGG ATC GCA AAG TTG
LIU523 (rev insert)	GTT TCC TGT GTG AAA TGC TTA GTA CAC AAT CAC TCT ACC AC
LIU524 (fwd vector)	ATT GTG TAC TAA GCA TTT CAC ACA GGA AAC AGC TAT GAC C
LIU525 (rev vector)	CCA GAT GAA GAA ATG AAT CAT CAC GCC ATG TAT CAG TGG
LIU126 (F0)	GCT GAT CGA CCC GCG CAT AC
LIU127 (R0)	CCA ATG ATC CAC AAT GGG TGA ATG C
Cloning of V. cholerae PmtlS500-*lacZ*(*Ec*)	
LIU136 (fwd insert)	CAT GGC GTG ATG ATT CTC CAG CCG CTA ATG CGC C
LIU130 (rev insert)	TGT TTC CTG TGT GAA ACA ACG GGG GAC GCG ATG ATA TC
LIU131 (fwd vector)	ATC GCG TCC CCC GTT GTT TCA CAC AGG AAA CAG CTA TGA CCA TG
LIU137 (rev vector)	CAT TAG CGG CTG GAG AAT CAT CAC GCC ATG TAT CAG TGG AC
LIU126 (F0)	See above
LIU127 (R0)	See above
Cloning of V. cholerae P*mtlA*_CRPbs	
LIU481 (F1)	GCC AAG CTT GCA TGC CTC CTC TCT TCG TGT ACC GC
LIU482 (R1)	TTT TTT GTG ACT TAC TTT GAT TTC TTG GTG ATC GGC ATT ATC
LIU483 (F2)	CAC CAA GAA ATC AAA GTA AGT CAC AAA AAA CCC GTT GGT G
LIU484 (R2)	AGT GAA TTC GAG CTC CCA ACA TTT CAA AGC CAC TGC GC
LIU485 (pCVD_F)	GCT TTG AAA TGT TGG GAG CTC GAA TTC ACT GGC CGT
LIU486 (pCVD_R)	ACA CGA AGA GAG GAG GCA TGC AAG CTT GGC GTA ATC ATG
LIU487 (F0)	GTG TAG GTC TTC CTA CTT ACG TAT AG
LIU377 (R0)	GAC CTG TTT CAC TGG CTT GCT G
Cloning of V. cholerae PmtlA_−10mut	
LIU481 (F1)	See above
LIU488 (R1)	CCC ACC ACA CAA ATT TCG AAT GGA ATC ACC AAC GGG TTT TTT G
LIU489 (F2)	GGT GAT TCC ATT CGA AAT TTG TGT GGT GGG GTG ATT GTG TAC
LIU484 (R2)	See above
LIU485 (pCVD_F)	See above
LIU486 (pCVD_R)	See above
LIU490 (F0)	GCT GCA TAA TCT AAA CGA GAT TCCA G
LIU377 (R0)	See above
Cloning of pmtlA5UTR	
LIU590 (fwd insert)	CTA CTG TTT GCT AGC GTA CTA AGC AAT CAA CGG TTT TTG CC
LIU591 (rev insert)	AAA ACA GCC AAG CTT CGC GTC CCC CGT TGG ATG TTC CG
LIU592 (rev vector)	GCT AGC AAA CAG TAG AGA GTT GCG
LIU593 (fwd vector)	AAG CTT GGC TGT TTT GGC GGA TG

a fwd, forward; rev, reverse; FW, forward; RV, reverse.

b Underlined regions indicate homology tails for fragment ligation using DNA fragment assembly. IRD800, IRdye 800 (Integrated DNA Technologies).

V. cholerae strains were struck out on Luria-Bertani (LB) plates with the appropriate antibiotics for 12 to 16 h at 37°C. For liquid cultures, individual colonies were grown for 12 to 16 h in 2 ml of LB or 1× M9 minimal medium containing one or more carbon sources (0.4% [wt/vol] each) and supplemented with 0.1% (wt/vol) trace metals (5% MgSO_4_, 0.5% MnCl_2_, 0.5% FeCl_3_, 0.4% nitrilotriacetic acid). Antibiotics were used at the following concentrations: streptomycin (Sm) at 100 μg/ml and carbenicillin (Cb) at 50 to 100 μg/ml. Transformation of V. cholerae strains was performed using plasmids originally propagated in E. coli TOP10 cells (except for the pCVD442-based plasmids [see below]). Plasmid pmtlA5UTR was constructed using primers LIU590 to LIU593 and a DNA fragment assembly using Hi-Fi master mix (New England BioLabs [NEB]).

V. cholerae strains harboring chromosomal mutations were constructed as follows: a plasmid bearing the desired mutation (including point mutations or deletions) was constructed in the allelic exchange vector pCVD442 via splicing by overlap extension (SOE) PCR. Two 500- and 650-bp DNA fragments flanking the region of interest were amplified by PCR using the F1/R1 and F2/R2 primer pairs (Table 2). These fragments were annealed together and then amplified by PCR using the F1 and R2 primers. The final PCR product was assembled via Hi-Fi DNA assembly (New England BioLabs) with the pCVD442 backbone, which was prepared using the appropriate pCVD_F and pCVD_R primers (Table 2). The resultant plasmid was propagated in E. coli DH5αλpir and transformed into E. coli SM10λpir before being conjugated into V. cholerae. Successful conjugates were selected from one round of growth in LB broth with streptomycin, and the resultant colonies were plated on sucrose medium to screen for successful vector disintegration. Sucrose-resistant colonies were screened for the desired mutation by PCR with the F0 and R0 primers.

To assemble the *lacZ* transcriptional fusion reporters, we first constructed a V. cholerae strain with a deletion in the promoter region (235 bp upstream) of VC2338, the V. cholerae homologue of *lacZ*. This was done to render the VC2338 locus inert, as the locus is prone to regulation by transcription factors, such as CRP-cAMP. The ribosome-binding site (RBS) and coding sequence of E. coli *lacZ* [*lacZ*(*Ec*)] were then cloned into pCVD442 derivative pJL1 using primers LIU122, LIU123, LIU124, and LIU125 and DNA fragment assembly using the Hi-Fi master mix (NEB). pJL1 contains an internal fragment of VC2338 which allowed *lacZ*(*Ec*) to be inserted into the VC2338 locus in the antisense orientation. We then fused the 500 bp directly upstream of the +1 site relative to either *mtlA* or *mtlS* transcription to the site immediately preceding the RBS of *lacZ*(*Ec*) using the chromosomal mutation method described above.

### LacZ (beta-galactosidase) assay.

All LacZ assays were performed using strains containing a *lacZ* gene construct that was inserted into the endogenous *lacZ* gene in order to disrupt native *lacZ* expression. Bacterial samples were taken from back-diluted liquid cultures grown to late log phase (optical density at 600 nm [OD_600_], 1.0 to 1.5). Cell samples (200 μl) were loaded onto a clear 96-well plate, and OD_600_ measurements were taken using a Synergy 4 plate reader (BioTek). From these samples, 100 μl of cells was lysed for 25 to 35 min with a 10-μl solution containing PopCulture reagent (Novagen) and lysozyme (Thermo Fisher) in a 1,000:1 ratio. Samples (30 μl) of cell lysate were then incubated with 150 μl of *o*-nitrophenyl-β-d-galactopyranoside (ONPG) substrate solution (60 mM Na_2_HPO_4_, 40 mM NaH_2_PO_4_, 1 mg/ml ONPG, 2.7 μl/ml β-mercaptoethanol) in a 96-well plate at 28°C. The absorbance at 420 nm (OD_420_) was recorded every 30 s over 60 min by a Synergy 4 plate reader (BioTek). Final results were reported as the average slope (in mean OD_420_ per minute) of the 30-s intervals over the course of the 60-min incubation period, with the units reported as the LacZ activity (mean OD_420_ per minute per OD_600_). Statistical analysis was performed using GraphPad Prism (version 7) software.

### Western blot analysis.

Cell pellets were prepared from back-diluted liquid cultures grown to mid-log phase (OD_600_, ∼0.3). Following centrifugation at 8,000 × *g* for 5 min at 4°C, pellets were resuspended in M9 medium, mixed 1:4 in SDS sample buffer (250 mM Tris-HCl [pH 6.8], 10% SDS, 50% glycerol, 10% β-mercaptoethanol, 0.5% orange G) and heated at 95°C for 10 min. Samples were loaded onto an SDS-containing 10% Tris gel (Bio-Rad) and run at 200 V for 30 min. Proteins were then transferred to a nitrocellulose membrane using a TransBlot Turbo transfer system (7 min at 1.3 A; Bio-Rad). Membranes were incubated with a dilution of primary antibody (1:5,000 of both rabbit anti-FLAG [AbCam] and mouse anti-RNAPα [AbCam]) for 1 h, followed by incubation with a dilution of secondary antibody (1:7,500 of both IR680-conjugated goat anti-rabbit immunoglobulin [LI-COR] and IR800-conjugated goat anti-mouse immunoglobulin [LI-COR]) for 30 min. Infrared fluorescence imaging was conducted using an Odyssey imager (LI-COR), and quantification of blots was performed with ImageStudio (version 5) software (LI-COR).

### RNA isolation.

To measure the mRNA levels of MtlS sRNA, total RNA was isolated from a bacterial culture grown to mid-log phase using a DirectZol RNA miniprep kit (Zymo). For half-life experiments, rifampin (200 to 300 μg/ml) was added upon cells reaching mid-log growth, and samples were extracted at the indicated time points. Following centrifugation (5,000 × *g*, 5 min, 4°C), the pellets were resuspended in TRI Reagent. Manufacturer instructions were then followed to isolate RNA, with column elution being performed in DNase- and RNase-free ultrapure water. For the qRT-PCR experiments, the remaining DNA was removed from all samples using a Turbo DNA-free kit (Thermo Fisher Scientific), according to the manufacturer’s suggested protocol. RNA concentrations were measured using a Take3 plate (BioTek).

### *In vitro* RNA preparation.

To construct the biotinylated RNA riboprobes, a DNA template was first prepared in the following PCR mixture: 200 μM deoxynucleoside triphosphates, 1 μM forward primer, 1 μM reverse primer, genomic DNA from V. cholerae strain JL2, 1× buffer, and *Taq* DNA polymerase (NEB). The DNA template was then used in an *in vitro* transcription assay performed with T7 RNA polymerase according to the manufacturer’s instructions: 0.5 mM each of ATP, CTP, and GTP; 0.3 mM UTP; 0.2 mM biotin-16-UTP; 10 μM dithiothreitol; DNA template; 1× buffer; and T7 RNA polymerase (Promega). The reaction mixture was allowed to incubate at 37°C for 1 to 3 h prior to addition of and incubation with RQ1 DNase at 37°C for 30 min. The riboprobe was purified using a Micro P-30 column (Bio-Rad).

### Northern blot analysis.

To prepare samples for Northern blotting, total RNA was mixed 1:2 in Loading Buffer II (Life Technologies). RNA was separated on a 10% Tris-borate-EDTA (TBE)–urea gel, run at 200 V for 50 to 60 min in 1× TBE. Transfer to a positively charged nylon membrane was performed using the TransBlot Turbo transfer system (7 min at 1.3 A; Bio-Rad).

Following a wash in 6× saline sodium citrate (SSC) for 2 min, the nylon membrane was subjected to UV cross-linking, followed by another wash in 1× SSC for 1 min. The membrane was then prehybridized for at least 30 min in ULTRAhyb-Oligo buffer (Life Technologies) at 65°C. Overnight hybridization was performed at 65°C with the appropriate riboprobe and a 5S DNA probe (IR800-5S). The membrane was subsequently washed two times for 5 min each time and two times for 15 min each time in low- and high-stringency wash buffer, respectively, according to the Odyssey Northern blot analysis protocol instructions (LI-COR). Fluorescence imaging was conducted using the Odyssey imager (LI-COR). Band quantifications were performed using ImageStudio (version 5.0) software (LI-COR). Statistical analysis was performed using GraphPad Prism (version 7) software.

### qRT-PCR.

RNA samples were used for quantitative reverse transcription-PCR (qRT-PCR) to quantify relative expression levels using a Stratagene MX3005P system, a Brilliant II SYBR green qRT-PCR master mix kit (Agilent), and primers specific to *mtlA*, *mtlS*, and 4.5S RNA. The reaction mixtures were set up in 96-well optical reaction plates and contained 1× Brilliant SYBR green qPCR master mix, 30 nM carboxy-X-rhodamine reference dye, each primer at 100 nM, 100 ng RNA, and 1 μl reverse transcriptase-RNase block enzyme mixture in a 25-μl reaction mixture. The following conditions were used for cDNA synthesis and PCR: 30 min at 50°C, 10 min at 95°C, and 40 cycles of 30 s at 95°C and 1 min at 60°C (Agilent). MxPro QPCR software (version 4.10) was used to determine the threshold cycle (*C_T_*) values for each reaction, and relative RNA concentrations were calculated from the *C_T_* values by comparison to standard curves. All transcript levels were normalized to a 4.5S RNA endogenous control. No signals were detected in the no-template controls and no-reverse transcriptase controls. Statistical analysis was performed using GraphPad Prism (version 7) software.
